# Relationship between Bronchial Hyperresponsiveness and Impaired Lung Function after Infantile Asthma

**DOI:** 10.1371/journal.pone.0001180

**Published:** 2007-11-14

**Authors:** Christophe Delacourt, Marie-Rose Benoist, Muriel Le Bourgeois, Serge Waernessyckle, Patrick Rufin, Jean-Jacques Brouard, Jacques de Blic, Pierre Scheinmann

**Affiliations:** Laboratoire d'Explorations Fonctionnelles Respiratoires, Service de Pneumologie et Allergologie Pédiatriques, Hôpital des Enfants Malades, Paris, France; Oregon Health & Science University, United States of America

## Abstract

Wheezing during infancy has been linked to early loss of pulmonary function. We prospectively investigated the relation between bronchial hyperresponsiveness (BHR) and progressive impairment of pulmonary function in a cohort of asthmatic infants followed until age 9 years. We studied 129 infants who had had at least three episodes of wheezing. Physical examinations, baseline lung function tests and methacholine challenge tests were scheduled at ages 16 months and 5, 7 and 9 years. Eighty-three children completed follow-up. Twenty-four (29%) infants had wheezing that persisted at 9 years of age. Clinical outcome at age 9 years was significantly predicted by symptoms at 5 years of age and by parental atopy. Specific airway resistance (sRaw) was altered in persistent wheezers as early as 5 years of age, and did not change thereafter. Ninety-five per cent of the children still responded to methacholine at the end of follow-up. The degree of BHR at 9 years was significantly related to current clinical status, baseline lung function, and parental atopy. BHR at 16 months and 5 years of age did not predict persistent wheezing between 5 and 9 years of age, or the final degree of BHR, but it did predict altered lung function. Wheezing that persists from infancy to 9 years of age is associated with BHR and to impaired lung function. BHR itself is predictive of impaired lung function in children, strongly pointing to early airway remodeling in infantile asthma.

## Introduction

Many infants wheeze during acute lower respiratory tract illness, but the wheezing usually ceases after 3 years of age [1]. Respiratory events occurring during the first years of life, even when transient, may influence the expression of asthma and lung function during childhood and early adulthood [2]. Children who wheeze from infancy to 6 years of age are at a high risk of long-term persistent wheezing [2]. Infantile wheeze was also recently linked to early signs of airway obstruction [2], [3]. Identification of factors predictive of clinical or functional deterioration in infants who wheeze would allow appropriate treatment to be started early. The role of bronchial hyperresponsiveness in these early alterations of lung function has rarely been investigated. It has been postulated that nonspecific bronchial hyperresponsiveness is a risk factor for accelerated pulmonary function decline during aging and for the onset of chronic airway obstruction [4]–[6]. More recently, Palmer and coworkers showed that early airway hyperresponsiveness was associated with a decreased baseline FEV1 by school age [7]. We have previously found that bronchial hyperresponsiveness persisted at 5 years of age in all children with infantile asthma, defined by three or more wheezing episodes before 2 years of age [8]. However, asthmatic infants who persisted to wheeze at 5 years of age had significantly stronger bronchial hyperresponsiveness to methacholine than children who became asymptomatic [9]. Persistent airway hyperresponsiveness may be therefore a significant contributor both to persistent symptoms and to airway obstruction. The children who participated in our first study were evaluated here for lung function and bronchial hyperreactivity at seven and nine years of age. We found that persistent infantile asthma was associated with an early increase in airway responsiveness, which likely contributed to impaired pulmonary function.

## Methods

### Subjects and Study Design

The study was approved by the ethics committee of Necker-Enfants Malades Hospital, and informed consent was obtained from the parents of all the children.

The cohort on which this study was based has been described in detail elsewhere [9]. Briefly, 129 infants who had had at least three episodes of wheezing were recruited at the Paediatric Chest Unit of Necker-Enfants Malades Hospital in Paris. Their mean age (±standard deviation) at enrolment was 16±7 months (range 11 to 24 mo). One hundred and twelve of these children were evaluated 4 years later, and results were previously published [9]. After this evaluation at 5 years of age, the children had a physical examination, lung function tests and a methacholine challenge test every 2 years until the age of 9 years. Eighty-three children (57 boys, 26 girls) had all the evaluations and formed the basis for the present study. At each evaluation, children with wheezing during the past year were classified as symptomatic, whereas those with no respiratory symptoms during the past year were stated as asymptomatic. Children were skin prick tested at 9 years of age, for at least 6 local aeroallergens, including *Dermatophagoides pteronyssinus* and *farinae,* cat, dog, birch and grass pollen (Stallergene SA, Antony, France). Personal atopy was defined as one or more tests producing a wheal of 3 mm or more [10]. The child's mother and/or father was atopic (asthma, allergic rhinitis or atopic eczema) in 44% of cases.

### Lung Function Tests

All the children had to be asymptomatic for at least 1 week before the day of the test, which was otherwise rescheduled.

Forced vital capacity (FVC, liters) and forced expiratory volume in one second (FEV1, liters) were measured at age 9 years by using a Sensormedics 2400 spirometer (SEBAC-MSR, Pantin, France).

Specific airway resistance (sRaw) was measured at ages 5, 7 and 9 years by using a whole-body plethysmograph (M. Autobox, SEBAC-MSR, Pantin, France). SRaw was calculated by simple algebraic manipulation of the known formulas for airway resistance and total gas volume [11]. This precludes the need for shutter occlusion, because sRaw is derived directly from the relationship between plethysmographic volume and respiratory flow. Measurements were made with a mouthpiece and nose clip. Three measurements of sRaw were made, and each was calculated from the medians of five consecutively measured technically acceptable loops [12]. The mean of these three sRaw measurements was used in the analysis. Because of methacholine challenge, post-bronchodilator values were not obtained.

### Methacholine Challenge

The methacholine aerosol was administered with a dosimeter (MFDC 88, Mediprom, Paris, France) attached to a nebulizer (De Vilbis 5610 D; particle size 1.9 µm MMAD). The apparatus was programmed to deliver a dose of 50 µg of methacholine in 40 ml of air in 0.5 s. In these conditions the duration and volume of each aerosol dose did not exceed the inspiratory time or volume, so each delivered dose was completely inhaled. The dosimeter was triggered by the inspiratory negative buccal pressure. The children initially inhaled normal saline. SRaw was measured two minutes later. This sequence was repeated after data collection. The initial methacholine dose was 50 µg and the dose was doubled for each sequence until sRaw increased by at least 100% from baseline or until a maximal methacholine dose of 1600 µg was inhaled. The dose provoking a doubling of sRaw was derived from the plot of the log methacholine dose against sRaw, by linear interpolation between the last two points on the semilogarithmic dose–response graph. Children who did not respond to the maximal dose of methacholine were assigned the value PD100 = 2400 µg (i.e., one half the maximal dose). At 16 months of age, the response to methacholine was evaluated by the fall in transcutaneous oxygen tension, and a 15% fall was considered as a positive response (PD15Ptc02) [9].

### Data Analysis

At each evaluation, children with wheezing during the past year were classified as symptomatic, whereas those with no respiratory symptoms during the past year were stated as asymptomatic. At the end of the 9-year follow-up period the children were classified into three groups:

- persistent wheezers: children classified as symptomatic at all three evaluations
- intermittent wheezers: children intermittently classified as symptomatic
- asymptomatic children: children classified as asymptomatic at all three evaluations.

All data are expressed as the mean±standard deviation (SD). The Kolmogorov-Smirnov test was applied to test for a normal distribution in all parameters. At each evaluation, sRaw values were independent of weight and height and were expressed as cmH_2_O.s. FEV1 was expressed as a percentage of FVC (FEV1/FVC). Height adjusted-V'max FRC values at age 16 months were converted to z scores. PD15PtcO2 and PD100sRaw values were log-transformed before analysis. For comparisons between time points, log values were converted to z scores to make them comparable. Linear regression and the chi2 test were used for comparisons between continuous and qualitative variables, respectively. Analysis of variance (ANOVA) followed by Fisher's PLSD test was used to test for intergroup differences. ANOVA with repeated measures was used for between-group comparisons over time. Odds ratios were obtained by logistic regression analysis. P values<0.05 were considered to denote significant differences.

## Results

One hundred and twenty-nine infants aged 16 months were enrolled in this prospective study. One hundred and twelve of these children were evaluated 4 years later and 83 attended the final visit at age 9 years. Their mean age±SD was 9.3±0.7 years (range 8.1–10.9). Mean anthropometric data at each visit are summarized in Table 1. Children lost to follow-up between 5 and 9 years of age did not differ from children who completed the study (Table 1).

**Table 1:** Characteristics of children who completed the study and those who were lost to follow-up between 5 and 9 years of age.

	Children who completed the study (n = 83)	Children lost to follow-up between 5 and 9 years (n = 29)
Age at final visit	9.3±0.7	-
Sex (% male)	68	67
% with parental atopy	44	45
% asymptomatic at 5 years	63	57
sRaw at 5 years (kPa/s)	7.9±2.2	7.5±2.9
PD100 sRaw at 5 years (log)	2.413±0.374	2.249±0.563

No significant difference was observed between the groups.

### Clinical Progression

**Figure 1:**
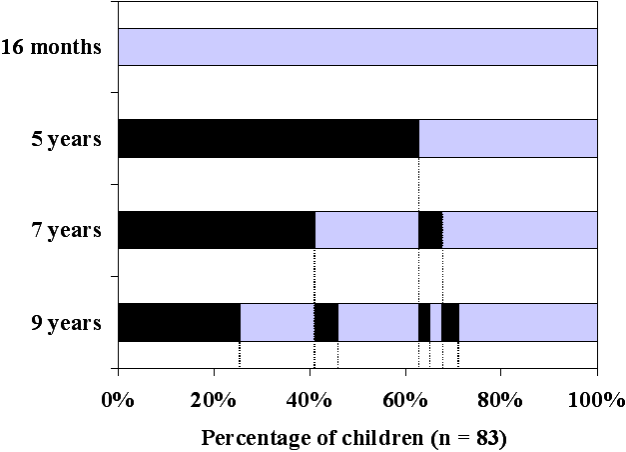
Clinical progression in 83 children followed from inclusion to 9 years of age. At each evaluation, children were divided into symptomatic (gray part of bars) and asymptomatic (black part of bars). Each bar represents 100% of children who completed the study (n = 83). Twenty-nine percent of children were classified as symptomatic at 5 years, 7 years, and 9 years, and were classified as persistent wheezers. Forty-six percent of children were intermittently classified as symptomatic (intermittent wheezers), and 25% of children were classified as asymptomatic at all three evaluations (asymptomatic children).

Although the majority of included infants were in apparent remission at 5 years of age, the rate of children with wheezing increased with age (Figure 1). Symptomatic children were 37%, 54%, and 64% at 5, 7, and 9 years of age, respectively. Twenty-four (29%) of the 83 children examined were categorized as persistent wheezers, 38 (46%) as intermittent wheezers, and 21 (25%) as asymptomatic. Thirteen children (16%) were taking inhaled corticosteroids (IC) at 9 years. The majority (69%) of children with IC had persistent symptoms. The remaining 31% had intermittent symptoms. Among the 62 children who wheezed between 5 and 9 years of age, 42 children experienced at least one episode of breathlessness requiring oral steroids or an emergency visit, while 20 children had two or more such episodes. Being symptomatic at 9 years of age was significantly predicted by being symptomatic at 5 years of age (OR = 4.8 (95% CI: 1.6–14.5); p<0.006) and by parental atopy (OR = 3.1 (95% CI: 1.1–8.2); p<0.03).

Skin prick tests were performed in 74 children, of whom 24 (32%) reacted to at least one allergen. No significant association was found with clinical findings at 9 years.

### Baseline lung function

**Figure 2:**
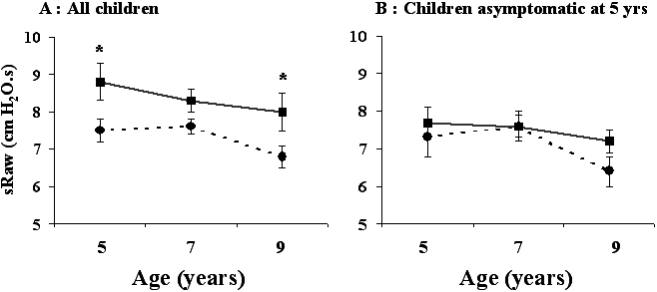
Mean sRaw values (±SEM) at each visit. A: Children were divided into 2 subgroups according to their clinical status at 5 years: asymptomatic (n = 52, circles and dashed line), and symptomatic (n = 31, squares and solid line). B. Asymptomatic children at 5 years were divided into 2 subgroups according to their clinical status at 9 years: asymptomatic (n = 25, circles and dashed line), and symptomatic (n = 27, squares and solid line). * : significant difference when compared to the asymptomatic group (p<0.05).

Baseline sRaw was evaluated at ages 5, 7, and 9, whereas spirometry was performed only at age 9 years. At age 9 years, mean sRaw, FEV1, and FEV1/FVC values were 7.3±2.3 cmH20.s, 95.8±13.8% of predicted, and 84.7±7.7%, respectively. The results of univariate analysis of factors potentially associated with baseline lung function at 9 years of age are shown in Table 2. Previous lung function results and the clinical history, but not current clinical status, were significantly related to lung function results at age 9 years. In particular, symptomatic children at 5 years of age had significantly higher sRaw levels and lower FEV1/FVC values at age 9 years. SRaw levels were already elevated at age 5 years in these children, and did not change thereafter (Figure 2A). Children who were asymptomatic at age 5 years continued to have low sRaw values, independently of their clinical outcome at 9 years of age (Figure 2B).

**Table 2:** Univariate analysis of factors potentially associated with sRaw and FEV1/FVC values at 9 years of age.

	SRaw (cmH20.s)	FEV1/FVC (%)
**VmaxFRC (Z score) at 16 months**	r = −0.431; p = 0.0002	NS
**sRaw (cmH20.s) at 5 years**	r = 0.371; p = 0.0024	r = −0.352; p = 0.0043
**sRaw (cmH20.s) at 7 years**	r = 0.549; p<0.0001	r = −0.403; p = 0.0006
**Overall clinical classification**		
Asymptomatic (n = 21)	6.1±1.9	86.6±7.5
Intermittent (n = 38)	7.5±1.7*	86.3±6.2
Persistent (n = 24)	7.8±3.3*	81.4±9.0*
**Clinical status at 5 years**		
Asymptomatic (n = 52)	6.8±1.8	86.8±6.4
Symptomatic (n = 31)	8.0±3.0*	81.8±8.8*
**Clinical status at 7 years**		
Asymptomatic (n = 38)	6.6±1.8	86.1±7.3
Symptomatic (n = 45)	7.8±2.7*	83.8±8.1
**Clinical status at 9 years**		
Asymptomatic (n = 30)	6.7±0.4	86.6±7.3
Symptomatic (n = 53)	7.5±0.4	83.9±7.9
**Number of severe exacerbations between ages 5 and 9 years**		
None (n = 42)	6.7±2.0	86.3±6.9
One (n = 20)	7.4±1.6	86.5±6.7
Two or more (n = 18)	8.4±3.4*	79.9±8.8*
**Parental atopy**		
Absence (n = 42)	7.1±1.9	86.1±7.5
Presence (n = 36)	7.4±2.9	83.3±8.0
**Personal atopy**		
No positive skin tests (n = 49)	7.5±2.3	85.1±7.6
≥1positive skin test (n = 24)	6.8±2.6	83.7±8.7

Severe exacerbations were defined by the need for oral steroids or an emergency visit. Parental atopy was defined by the diagnosis of asthma or allergic rhinitis in the mother or father. Linear regression was used to compare continuous variables, and analysis of variance to compare groups. * p<0.05 when compared to reference group.

### Airway reactivity to methacholine

Only 5% of the 9-year-old children tested did not respond to methacholine, up to a dose of 1600 µg. We first analyzed factors associated with PD100 sRaw values at 9 years of age (Table 3). Lower PD100 sRaw values at 9 years were significantly related to persistent symptoms from age 5 years to 9 years, and also with a larger number of severe exacerbations between 5 and 9 years, parental atopy, and an obstructive lung function pattern at age 9 years. By contrast, PD100 sRaw at age 9 years was not predicted by the degree of airway hyperresponsiveness at 16 months or 5 years of age, suggesting that hyperresponsiveness increased gradually in asthmatic infants who continued to wheeze. To confirm that the relationships described are not dependant upon the presence of the four children who did not respond to methacholine, and who were assigned an arbitrary high value, we re-analyzed data on the 79 responder children (Table 3). Significant results were confirmed, except for the relationship between PD100 sRaw values at 9 years of age and clinical status at 7 years (p = 0.0882).

**Table 3:** Univariate analysis of factors associated with PD100 sRaw values at 9 years of age.

	PD100 sRaw (log) at 9 years	
	All children (n = 83)	Responder children (n = 79)
**VmaxFRC (Z score) at 16 months**	r = 0.270; p = 0.0269	r = 0.264; p = 0.0310
**sRaw (cmH20.s) at 5 years**	NS	r = −0.322; p = 0.0130
**sRaw (cmH20.s) at 9 years**	r = −0.266; p = 0.0203	r = −0.323; p = 0.0053
**FEV1/FVC (%) at 9 years**	r = 0.382; p = 0.0007	r = 0.375; p = 0.0010
PD15 PtcO2 (log) at 16 months	NS	NS
PD100 sRaw (log) at 5 years	NS	r = 0.361; p = 0.0017
**PD100 sRaw (log) at 7 years**	r = 0.429; p = 0.0002	r = 0.501; p<0.0001
**Overall clinical classification**		
Asymptomatic (n = 21)	2.568±0.342	2.568±0.342
Intermittent (n = 38)	2.469±0.435	2.355±0.305*
Persistent (n = 24)	2.245±0.260*	2.245±0.260*
**Clinical status at 5 years**		
Asymptomatic (n = 52)	2.530±0.389	2.476±0.333
Symptomatic (n = 31)	2.266±0.323*	2.225±0.243*
**Clinical status at 7 years**		
Asymptomatic (n = 38)	2.528±0.418	2.453±0.344
Symptomatic (n = 45)	2.348±0.342*	2.322±0.302
**Clinical status at 9 years**		
Asymptomatic (n = 30)	2.540±0.362	2.511±0.331
Symptomatic (n = 53)	2.370±0.390 (p<0.06)	2.303±0.297*
**Number of severe exacerbations between ages 5 and 9 years**	p = 0.0028 (ANOVA)	
None (n = 42)	2.503±0.381	2.466±0.333
One (n = 20)	2.540±0.409	2.441±0.299
Two or more (n = 18)	2.163±0.217*	2.154±0.217*
**Parental atopy**	p = 0.0293 (ANOVA)	
Absence (n = 42)	2.512±0.381	2.475±0.335
Presence (n = 36)	2.323±0.381*	2.291±0.290*
**Personal atopy**		
No positive skin tests (n = 49)	2.416±0.385	2.382±0.307
≥1 positive skin test (n = 24)	2.333±0.336 (NS)	2.285±0.253 (NS)

Data were analyzed with all children, and after exclusion of non responders to methacholine challenge. Severe exacerbations were defined as the need for oral steroids or an emergency visit. Parental atopy was defined by a diagnosis of asthma or allergic rhinitis in the mother or father. Linear regression was used to compare continuous variables, and analysis of variance to compare groups. * significance in post-hoc analysis.

**Figure 3:**
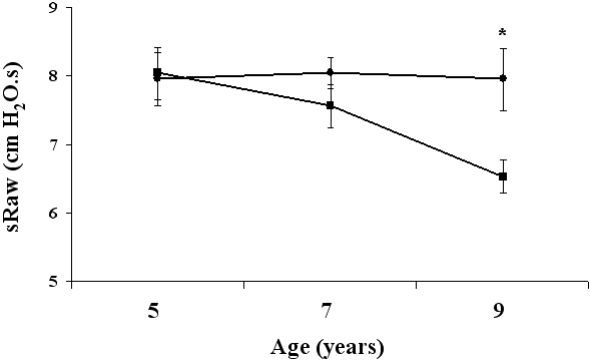
SRaw measurements at ages 5, 7 and 9 years according to the degree of bronchial hyperresponsiveness at age 5 years: children with PD100 sRaw below (circles) and above (squares) the median value of the population. *: p<0.008 between groups.

Persistent wheezing between 5 and 9 years of age was significantly associated with the degree of airway hyperresponsiveness at 7 and 9 years of age, but not with airway hyperresponsiveness at 16 months or 5 years (Table 4). Bronchial hyperresponsiveness was predictive of altered lung function. As early as 16 months of age, the degree of bronchial hyperresponsiveness correlated with higher sRaw values and lower FEV1/FVC values at 9 years of age (Table 4). The PD100 sRaw values at age 5 years significantly influenced sRaw values between 5 and 9 years (Figure 3). Among factors measured at 5 years, PD100 sRaw and sRaw were independently associated with higher sRaw values at age 9 years (Table 5).

**Table 4:** Relationship between the degree of bronchial hyperresponsiveness at 16 months and 5, 7 and 9 years of age, and the persistence of symptoms or lung function impairment at 9 years of age.

	OR (95% CI)	p value
**Risk = persistent wheezing between 5 and 9 years of age**		
PD15 PtCO2 (log) at 16 months	0.646 (0.214–1.955)	NS
PD100 sRaw (log) at 5 years	0.217 (0.038–1.243)	NS
PD100 sRaw (log) at 7 years	0.033 (0.004–0.262)	0.0013
PD100 sRaw (log) at 9 years	0.062 (0.008–0.487)	0.0082
**Risk = sRaw≥median value at age 9 years**		
PD15 PtCO2 (log) at 16 months	0.198 (0.074–0.531)	0.0013
PD100 sRaw (log) at 5 years	0.251 (0.070–0.897)	0.0334
PD100 sRaw (log) at 7 years	0.159 (0.040–0.626)	0.0085
PD100 sRaw (log) at 9 years	0.253 (0.071–0.903)	0.0342
**Risk = FEV1/FVC<median value at age 9 years**		
PD15 PtCO2 (log) at 16 months	0.402 (0.165–0.983)	0.0457
PD100 sRaw (log) at 5 years	0.907 (0.275–2.991)	NS
PD100 sRaw (log) at 7 years	0.132 (0.032–0.544)	0.0051
PD100 sRaw (log) at 9 years	0.027 (0.004–0.185)	0.0002

The risks were of being a persistent wheezer between 5 and 9 years of age, of having an sRaw value at or above the median value of the study population at 9 years of age, and of having an FEV1/FVC value below the median value of the population at 9 years of age. Median values were 7.0 cmH2O.s for sRaw and 85.5% for FEV1/FVC. Odds ratios were obtained by logistic regression.

**Table 5:** Multiple linear regression analysis of sRaw values at age 9 years.

	Regression coefficient	95% confidence interval	p value
sRaw (cm H20.s)	0.352	0.140 to 0.564	0.0015
PD100 sRaw (log)	−1.607	−2.878 to −0.336	0.0141
Clinical group	0.023	−1.048 to 1.095	0.9658

Parameters obtained at 5 years of age that correlated with 9-year sRaw values in univariate analysis were included as independent variables.

## Discussion

We investigated the relationship between bronchial hyperresponsiveness (BHR) and clinical status and lung function at 9 years of age in children who had infantile asthma. The mid-term results of this study, published elsewhere, showed that higher bronchial hyperresponsiveness was associated with persistent symptoms at 5 years of age [9]. Nevertheless, BHR persisted in infants who stopped wheezing. The present study, with a further four years of follow-up, sheds new light on the links between BHR and the persistence of symptoms and altered lung function in children who had infantile asthma. The main results are that: 1) most infants with recurrent wheezing still wheezed between 5 and 9 years of age; 2) clinical status at 5 years of age strongly influenced lung function at 9 years; and 3) bronchial hyperresponsiveness persisted in nearly all the children and was linked to progressive loss of lung function. One limitation of our study is to be unable to evaluate potential impact of anti-asthmatic treatments, and especially inhaled corticosteroids, on lung growth. Treatments delivered to children were not systematically collected. Only 16% of children received inhaled corticosteroids at 9 years of age.

### Clinical outcome of children with infantile asthma

All the infants enrolled in this study had recurrent wheezing at baseline. Four years later, at 5 years of age, only 37% continued to wheeze, with or without periods of remission, in keeping with epidemiological data [1], [13]. However, 75% of the children were still able to wheeze between 5 and 9 years of age, and nearly 30% had persistent wheezing. The symptoms were usually mild, as only one-quarter of the children experienced two or more episodes of breathlessness requiring oral steroids or an emergency visit. Nearly all the children who were still symptomatic at 5 years of age still wheezed at 9 years of age. Interestingly, we also found that most children who were in apparent remission at age 5 years had new wheezing episodes between the ages of 5 and 9. The persistent bronchial hyperresponsiveness that we previously observed in these infants is likely to explain this finding. Similarly to our findings, Morgan et al. found that around two-thirds of children with early and persistent wheezing were still symptomatic at ages 8 and 11 years, and that most of them had infrequent wheezing [2]. However, these authors found that only 20% of children with early and transient wheezing still wheezed at the age of 8 or 11 years [2]. This discrepancy is probably due mainly to differences in the study populations. All children in our study had infantile asthma, defined by three or more wheezing episodes before 2 years of age, whereas the infants in the Tucson study had only one or two wheezing episodes. A higher frequency of symptoms during infancy is a significant risk factor for the persistence of asthma at school age [14].

### Lung functional outcome in children with infantile asthma

SRaw and FEV1/FVC values at 9 years of age were influenced by clinical status at 5 years but not at 9 years. They were also predicted by sRaw values at 5 and 7 years of age. SRaw values at 9 years even correlated with VmaxFRC at 16 months of age. In particular, mean sRaw levels differed strongly between symptomatic and asymptomatic children at 5 years, and this difference remained stable thereafter, independently of clinical outcome. These results suggest that infantile asthma is a risk factor for early loss of lung function, which is visible by 5 years of age and may be irreversible thereafter. Our results support the findings of the Tucson study, in which forced expiratory flows were significantly lower among early and persistent wheezers than among late-onset wheezers [2]. Our results are also in keeping with those of Lowe and coworkers who showed that, by age 5 years, both transient and persistent wheezers have reduced lung function compared with non wheezers, and that the deficit is significantly greater in persistent wheezers [15]. Finally, prospective studies in which patients were followed up from school-age to adulthood showed that the lung function phenotype was acquired early in childhood and remained stable thereafter, regardless of the severity of asthma symptoms [16], [17]. The mechanisms that determine lower levels of lung function are controversial. It has been proposed that constitutional narrow airways predict subsequent wheezing illness throughout infancy [1], [18]. However, in our cohort of asthmatic infants we have previously shown that low Vmax FRC values are not significantly associated with transient wheezing but with persistent wheezing by the age of 5 years [9]. The low VmaxFRC values in our population are therefore likely to reflect disease severity rather than constitutionally small airways, exposing to persistence of symptoms and loss of lung function. Early airway remodeling may also contribute to reduced airway caliber [19]. Airway remodeling consists of structural changes associated with chronic airway inflammation [20]. Its severity is indirectly reflected by reduced expiratory flow rates and increased airway resistance, both of which are due to reduced airway caliber. Reduced airway caliber is also thought to play a role in persistent airway hyperresponsiveness to methacholine [21]. Our results support a link between increased airway resistance and higher BHR, and also a role of bronchial hyperresponsiveness in impaired lung function.

### Relationship between bronchial hyperresponsiveness and loss of lung function

Nearly all the children still responded to methacholine at 5 years of age, even those who were apparently in durable remission. This could contribute to symptom recurrence after a period of remission, as observed in our cohort between 5 and 9 years of age. Silent bronchial hyperresponsiveness increased the risk of wheezing and shortness of breath during follow-up among formerly asymptomatic patients [22]–[24]. Similarly, persistent BHR was found to significantly increase the risk of symptom relapse [17]. This underlying persistent component of BHR is thought to relate to airway remodeling [21]. It was previously suggested that this form of BHR detected in school children was a strong determinant of the future pattern of growth of airway function [20]. Our present study demonstrates that, as early as infancy, BHR itself is predictive of impaired lung function in children, strongly pointing to early airway remodeling in infantile asthma. Indeed, a high level of BHR at 16 months of age predicted reduced airway caliber at 9 years of age, as reflected by high sRaw and low FEV1/FVC values. In a recent study, no structural changes were observed by bronchial biopsy in wheezing infants [25]. The younger age and heterogeneity of the infants participating in this study may explain this apparent discrepancy. We also found that higher BHR at 5 years of age was significantly and independently associated with impaired growth of pulmonary function between 5 and 9 years of age. These findings are in line with previous reports. Indeed, FEV1 and BHR in asthmatic children were found to be independent predictors of FEV1 in adulthood [4]. Similarly, early airway hyperresponsiveness was associated with a decreased baseline FEV1 by school age [7]. Recently, Illi and coworkers found that BHR at 7 years of age was a significant predictor of baseline lung function at ages 7, 10, and 13 years, and suggested that BHR was the intermediate step in the causal pathway linking early-life sensitization to decrements in lung function at school age [26]. Neither parental nor personal atopy was linked to impaired lung function in our study. Similarly, Yang and coworkers found that pulmonary function in asthmatic children was not influenced by atopy, the serum IgE level or the total eosinophil count, but that it was significantly associated with the degree of BHR [27]. This suggests that factors other than atopy are involved in the onset of BHR in asthmatic infants.

The results of this study therefore support the hypothesis that persistent infantile asthma is associated with an early increase in airway responsiveness, and that this contributes to functional pulmonary impairment.
